# Modeling [Ca^2+^]_o_- and [K^+^]_o_-dependent oscillations in spinal Hb9 interneurons

**DOI:** 10.1186/1471-2202-13-S1-P49

**Published:** 2012-07-16

**Authors:** Natalia A Shevtsova, Sabrina Tazerart, Laurent Vinay, Frédéric Brocard, Ilya A Rybak

**Affiliations:** 1 Department of Neurobiology and Anatomy, Drexel University College of Medicine, Philadelphia, PA 19129, USA; 2 Institut de Neurosciences de la Timone (UMR7289), CNRS and Aix-Marseille Université, Marseille 13385, France; 3 Departments of Surgery and Anatomy and Neurobiology, Dalhousie University, Halifax NS B3H 3A7, Canada

## 

The spinal interneurons in newborn rodents, when synaptically isolated by removing the extracellular calcium ([Ca^2+^]_o_), demonstrate intrinsic rhythmic bursting activity that can be suppressed by riluzole, a blocker of the persistent sodium current (*I*_NaP_) [2]. This finding led to the suggestion that lowering of [Ca^2+^]_o_ may enhance *I*_NaP_ by shifting its activation threshold toward more negative voltages, and raised the question of functional relevance of this finding to generation of locomotor rhythm. To assess this issue, a series of experiments was performed *in vitro* using the isolated spinal cord preparation from the neonatal rat with measurements of [Ca^2+^]_o_ and extracellular potassium concentration ([K^+^]_o_) during pharmacologically induced fictive locomotion. We demonstrated that with the onset of fictive locomotion, [Ca^2+^]_o_ reduced from 1.2 up to 0.9 mM whereas [K^+^]_o_ increased from 4 up to 6 mM. At the same time, a special study performed on the isolated genetically identified Hb9 excitatory interneurons showed that, at [Ca^2+^]_o_= 1 mM and [K^+^]_o_=5 mM, 12% of Hb9 cells expressed intrinsic *I*_NaP_-dependent bursting, and at the concentrations typical for fictive locomotion ([Ca^2+^]_o_= 0.9 mM and [K^+^]_o_=6 mM), as many as 50% of identified Hb9 interneurons expressed *I*_NaP_-dependent bursting. Importantly, the threshold of [Ca^2+^]_o_ to generate bursting decreased as [K^+^]_o_ increased. The analysis of Hb9 neuron behavior during slow ramp increase of voltage revealed that lowering [Ca^2+^]_o_ from 1.2 to 0.9 mM induced a negative shift (~ -3 mV) in the *I*_NaP_ half-activation voltage (*V*_1/2NaP_). In contrast, *V*_1/2NaP_ was not changed when [K^+^]_o_ increased from 4 to 6 mM.

**Figure 1:**
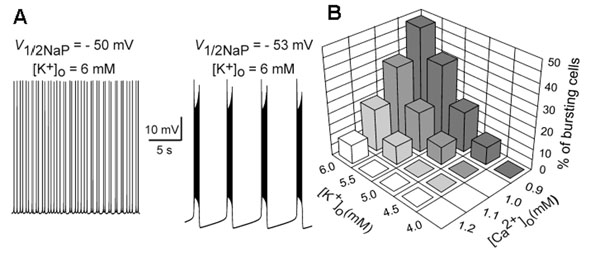
**A**. Switching from tonic to bursting activity in the modeled neuron by shifting *V*_1/2NaP_ to more negative value. **B**. *The synergistic effect of* [Ca^2+^]_o_ and [K^+^]_o_*changes on emergence of bursting activity* in the modeled population of 50 uncoupled cells.

To theoretically investigate the effect of changing [Ca^2+^]_o_ and [K^+^]_o_ on the Hb9’s pacemaker properties and firing behavior, we developed a single-compartment computational model of Hb9 neuron. In this model, we explicitly simulated a negative shift of *V*_1/2NaP_ occurring with the reduction of [Ca^2+^]_o._. At [K^+^]_o_=6 mM, our model exhibited tonic activity at *V*_1/2NaP_ = –50 mV (Fig. 1A, *left*). The rhythmic bursting emerged at *V*_1/2NaP_ = –51 mV, and further shifting *V*_1/2NaP_ to the left produced stable bursting (Fig. 1A, *right*). In turn, an increase in [K^+^]_o_ reduced the potassium reversal potential and hence all voltage-gated potassium currents (*I*_K_), which provided an additional augmentation of *I*_NaP_-dependent bursting [1]. To study *a synergistic effect of* [Ca^2+^]_o_ and [K^+^]_o_*on the emergence of bursting activity*, *we modeled* a population of 50 uncoupled neurons with randomly distributed parameters (see Fig. 1B). Our simulations have shown that shifting *V*_1/2NaP_ towards more negative values induced by reducing [Ca^2+^]_o_ may play a major role in emergence of bursting activity in the population of spinal interneurons. We have also demonstrated that accumulation of [K^+^]_o_*can* facilitate the emergence of *I*_NaP_-dependent bursting via the reduction of *I*_K_.

In summary we suggest that co-regulation of *I*_NaP_ and *I*_K_ by the corresponding changes in [Ca^2+^]_o_ and [K^+^]_o_ may convert activity of spinal interneurons from asynchronous/tonic to the synchronized bursting. This activity-dependent switching in firing behavior may represent a fundamental mechanism for locomotor rhythm generation in the spinal cord.
